# Effect of alcohol consumption on relapse outcomes among tuberculosis patients: A systematic review and meta-analysis

**DOI:** 10.3389/fpubh.2022.962809

**Published:** 2022-11-03

**Authors:** Dao Weiangkham, Adinat Umnuaypornlert, Surasak Saokaew, Samrerng Prommongkol, Jutamas Ponmark

**Affiliations:** ^1^Department of Nursing, School of Nursing, University of Phayao, Phayao, Thailand; ^2^Division of Social and Administration Pharmacy, Department of Pharmaceutical Care, School of Pharmaceutical Sciences, University of Phayao, Phayao, Thailand; ^3^Center of Health Outcomes Research and Therapeutic Safety (Cohorts), School of Pharmaceutical Sciences, University of Phayao, Phayao, Thailand; ^4^Unit of Excellence on Clinical Outcomes Research and IntegratioN (UNICORN), School of Pharmaceutical Sciences, University of Phayao, Phayao, Thailand; ^5^Mahidol Bangkok School of Tropical Medicine (Mahidol-BSTM), Faculty of Tropical Medicine, Mahidol University, Bangkok, Thailand

**Keywords:** tuberculosis relapse, alcohol abuse, alcoholic, health risks, tuberculosis

## Abstract

**Introduction:**

Tuberculosis (TB) is one of the major public health issues in every country. Alcohol consumption is one of the reasons associated with the severity of symptoms and death among TB patients. The impact of alcohol use on TB relapse outcomes is still debatable. This study aimed to conduct a systematic review and meta-analysis (SR/MA) to find the link between alcohol use and TB relapse outcomes.

**Methods:**

Data collection was performed from December 2021 to March 2022; and was obtained from electronic databases including CINAHL, PubMed, and Scopus. The researcher carefully searched and reviewed all the relevant research concerning drinking alcohol and relapse outcomes among TB patients. A set of inclusion and exclusion criteria was used to assess research publications. The methodological quality of eligible publications was assessed using the Newcastle–Ottawa Scale. Random meta-analysis was used to determine odds ratios (ORs) with a 95% confidence interval (CIs). The funnel plot, Begg's test, and Egger's test were employed to investigate publication bias.

**Results:**

There were a total of 2,113 studies found and reviewed, and eight publications were chosen for the analysis. It was found that among TB patients with a moderate appearance of heterogeneity, drinking alcohol increases the probability of relapse (OR = 3.64; 95% CI: 2.26–5.88, *p* < 0.001) and mortality (OR = 1.72; 95% CI: 1.40–2.12, *p* < 0.001). The funnel plot, Begg's test, and Egger's test all revealed that there was no indication of publication bias.

**Conclusions:**

Relapses and mortality among tuberculosis patients are considerably increased by alcohol drinking. More research into the causality of this link between the degree of alcohol use and the underlying processes is required.

**Systematic review registration:**

PROSPERO [CRD 42022295865].

## Introduction

Tuberculosis (TB) is a major public health concern in every country (1). TB is a contagious illness that is one of the top 10 causes of mortality globally; and the main cause of death due to a single infectious agent (2). According to the World Health Organization (WHO), geographically, the majority of TB cases in 2019 were found in regions of South-East Asia (44%), Africa (25%), and the Western Pacific (18%), with smaller shares in the Eastern Mediterranean (8.2%), the Americas (2.9%), and Europe (2.5%). The eight countries that accounted for two-thirds of the total TB globally were India (26%), Indonesia (8.5%), China (8.4%), the Philippines (6.0%), Pakistan (5.7%), Nigeria (4.4%), Bangladesh (3.6%), and South Africa (3.6%) (2).

At present, the global TB incidence rate is slightly declining, but the relapse rate has increased (2). Between 2017 and 2019, the global number of people reported in 70 countries showed that the rate of treatment for multidrug-resistant TB (MDR) or rifampicin-resistant TB (RR-TB) increased by 10% or more (2). In 2017, WHO estimated that there were 10.0 million new cases of TB, including 0.56 million RR-TB cases, and of this, 82% were MDR-TB (3). The problem of TB relapse remains a major problem in underdeveloped or low-income countries. This is because underdeveloped countries have limited public health resources care due to a shortage of health personnel, budget, and technology, resulting in poor management. These patients have difficult access to the health system, resulting in discontinuation of medication, consequential in TB relapse.

Mycobacterium tuberculosis, a bacillus, causes TB, which is disseminated when persons with TB exhale germs into the air (e.g., by coughing). The illness mainly affects the lungs (pulmonary TB), but it can affect other organs as well (1). Due to comorbidities, such as diabetes, hypertension, rheumatism, and HIV infection, RR-TB patients bear greater spiritual and financial burdens in the event of TB relapse, low treatment success, and longer treatment period, especially among elderly patients who have significantly reduced physical function (1–3).

Exogenous reinfections cause TB relapses, which contribute to the prevalence rate of up to 27%. Their significance stems from the possibility of the emergence of multidrug-resistant Mycobacterium TB strains (4). The relapse of TB affects many aspects, including the physical, psychological, and social impacts on the patient (3). The retreatment of the patient increases the chance of developing drug-resistant pathogens; which requires patients to have a more extended treatment period, discourages patients from treating themselves, and makes treatment intermittent. TB relapse causes death, increases hospital admissions, and economic damage. It is a burden for the country to pay for medical expenses. Furthermore, households afflicted by TB endure catastrophic expenses. Since 2015, 17 countries have conducted a nationwide study of costs faced by TB patients and their homes, and it showed that with a range of 19–83%, 49% of persons with TB and their households suffered catastrophic expenses (defined as total costs equivalent to >20% of yearly household income). The proportion was even higher for persons with drug-resistant TB, at 80% (range: 67–100%) (3).

Factors affecting the development of TB depend on the infection concentration at the time of infection, nutrition, poverty, and disease or exposure to certain substances or drugs that suppress the immune system, that is, HIV infection; all these factors are to be considered. There are various recognized risk factors for TB relapse, including treatment methods, treatment length, treatment consistency, alcohol usage, smoking, increasing age, and HIV infection (1–8). The socio-demographic, cultural, clinical, and behavioral aspects are continuously being studied.

The effects of alcohol consumption on TB relapse have been studied, but the results have been inconsistent due to a variety of factors. It was not concluded that alcohol use was identified as a risk factor or correlated. Many studies suggest that alcohol has an effect on TB recurrence. Drinking alcohol has been assumed to be the cause of TB relapse. Alcohol reduces the activity of many types of white blood cells, which are the body's mechanisms used to rid the body of TB before spreading to the point of being sick; and white blood cells function less if we drink alcohol, such as macrophage, CD 4+ lymphocyte, and CD 8+ lymphocyte in T cells (9). People who drink alcohol have lower immunity than normal people. In addition, drinking alcohol can impair its effectiveness because alcohol interferes with Isoniazid, the primary drug used to treat TB (9). TB patients have a chance to get TB again. Patients with TB relapses increase the likelihood of developing drug-resistant pathogens, require a more extended treatment period, discourage patients from treating themselves, and make treatment intermittent. However, other studies have found that drinking alcohol has no effect on TB relapse (10).

TB relapse affects all aspects of the health system, family, community, and nation. In current studies, it was found that many TB patients had alcohol consumption behaviors. This behavior may be a contributing factor to TB relapse. However, the correlations between TB relapse and alcohol consumption are unclear and have potential public-health implications. Therefore, the researcher is interested in applying systematic review and meta-analysis methods to analyze correlations between alcohol use and the relapse of TB. The research aims to determine the effect of alcohol consumption on tuberculosis relapse outcomes.

## Methods

### Protocol and registration

The Preferred Reporting Items for Systematic Reviews and Meta-analyses (PRISMA) statement was used to conduct this systematic review and meta-analysis (11). This research was registered with PROSPERO (Registration Number CRD 42022295865).

### Data sources and search strategy

CINAHL, PubMed, and Scopus databases were extensively searched to find relevant papers. Whenever possible, MeSH (Medical Subject Headings) was employed. We looked through bibliographic databases for relevant literature. The following keywords were used in the keyword search strategy: [Tuberculosis] AND [alcohol consumption OR alcohol drinking ^*^] AND [relapse OR reinfection OR recurrence OR retreatment OR failure ^*^] with slight adjustments depending on the database. There was no requirement for a study design or a language constraint. To prevent missing any papers, further searches were conducted in the reference lists of included research.

### Study selection

This study included all relevant studies that presented clinical features and epidemiological information on drinking among TB patients. We applied the PICO approach to generate precise questions and searching terms which included, P: population (patient with Tuberculosis), I: intervention/exposure group (alcohol consumption), C: comparison group (no alcohol consumption), and O: outcomes (primary outcome: tuberculosis relapse; secondary outcome: death). We considered all publications of any kind (randomized controlled trials and observational studies). Exclusions included animal studies, reviews, comments, editorials, expert opinions, letters, conferences, meeting abstracts, case reports, case series, systematic reviews, and meta-analyses. Studies that did not contain impact estimates or had inadequate data to determine effect estimates were also excluded. Papers with a clear connection to the alcohol industry were excluded.

### Outcomes measures

The primary outcome was TB patients' illness recurrence. Death among TB patients was a secondary result. The medical records as diagnosed and described by physicians were included in the phrase “disease relapse.” Any of the following criteria were used to determine relapse (12):

(I) Tuberculosis patients who are diagnosed and described by physicians.
(II) Relapse case, defined as a patient who has previously been treated for TB and has relapsed due to the same TB infection that caused the original TB disease, or reinfection: due to infection with a new strain after successful TB therapy.
(III) Death case, as patient with TB; patient death from Tuberculosis disease.

### Data extraction and quality assessment

Each title, abstract, and full-text publication was separately screened for potentially suitable research by two investigators (The first and the fifth). Disagreements were settled after consulting with a third investigator (The third). All of the data that had been retrieved was examined independently by two scientists (The first and the fifth). The same investigators extracted information from all possibly relevant publications. Setting, geography, design, sample size, participant characteristics (such as age and sex), details of intervention/exposure (drinking status; non-drinking), details of outcomes (disease severity; relapse and death), and the number of TB patients were all retrieved from each research. The Newcastle–Ottawa Scale was used to assess the quality of individual research (NOS) (13). The NOS assigns a maximum of 9 points, with a total score of ≥7 indicating good quality research.

### Statistical analysis

For each trial, we calculated the odds ratio (OR) and 95% confidence interval (CI) using the number of drinkers and non-drinkers and pre-specified outcomes (relapse and death). A random-effects model was used to integrate the pool effects. Cochran's *Q*-statistic was used to study heterogeneity. To indicate heterogeneity among trials for each analysis, an alpha value of 0.10 was selected. *I*^2^ (*I* square) values were used to depict the degree of heterogeneity. High, moderate, and low heterogeneity were indicated by *I*^2^ values of more than 75%, 25–75%, and <25%, respectively (13). When there was heterogeneity, an attempt was made to investigate the probable origins of heterogeneity. Begg's test, Egger's test, and the funnel plot were used to assess publication bias (14–16). In publication bias tests, a *p* < 0.05 indicated publication bias (17).

### Sensitivity and subgroup analysis

When studies without adjusted OR were not reported, sensitivity analysis was used to assess the robustness of our analysis. Subgroup analyses were undertaken based on age, current and non-drinking status, and study quality.

## Results

**Figure 1:**
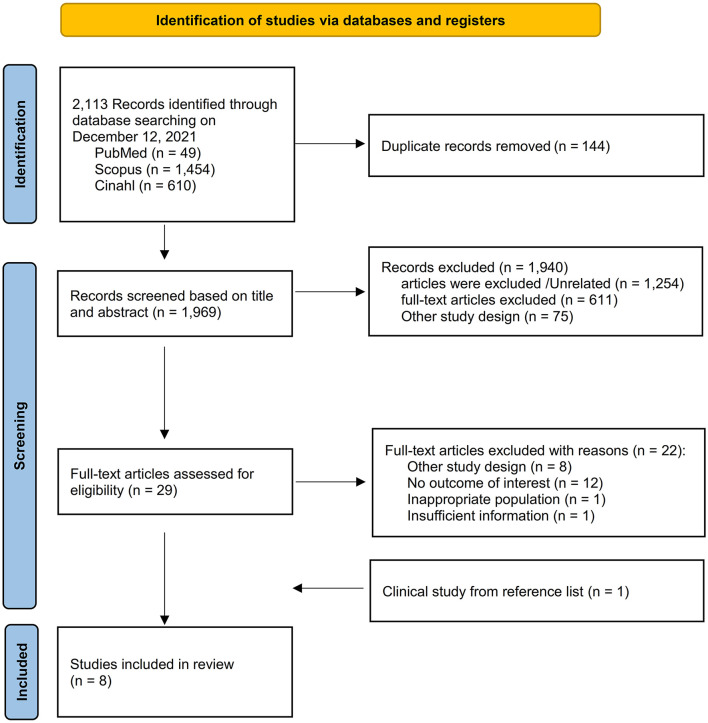
Selection flow diagram.

A total of 2,113 items were found in all databases during the first search. Duplicates were detected in 144 of these, which were removed. The title and abstract were used to filter all articles. After reviewing the abstracts, 1,940 papers were ruled out since their findings were unrelated to our goal. A total of eight papers comprising 17,128 TB patients were included in the meta-analysis after the complete text was evaluated (Figure 1). The important characteristics and outcomes of the included articles were collated (Table 1). Eight articles were conducted in China (3), one each in Columbia (4), Russia (18), India (19), Mexico (20), Croatia (7), South Africa (10), and Brazil (21). The majority of the publications were prospective studies. The average age of the patients in the studies considered was 50.28 years. Eight studies were included in the study: Four studies defined outcomes as relapse (3, 4, 7, 10) and four studies defined outcomes as death (18–21).

**Table 1:** General characteristics of eight studied included.

**References**	**Location**	**Study design**	**Baseline participant characteristics**		**Type of seeker**	**Outcomes measures**	**OR (95% CI)**	**Quality of study**
			**Participants**	**Age (years)**				
Chen et al. (3)	China	Retrospective cohort study	617	74.5	Alcohol	Relapse	3.57 (1.29–10.46)	8
Córdoba et al. (4)	Columbia	Case control study	162	51.55	Alcoholic	Relapse	5.56 (1.18–26.26)	8
Kurbatova et al. (18)	Russia, Latvia, and Estonia	Retrospective cohort study	823	36	Alcohol	Death	1.69 (1.09–2.61)	5
Cox et al. (19)	India	Prospective cohort study	751	39	Alcohol	Death	1.9 (1.08–3.34)	8
Abdelbary et al. (20)	Mexico	Case control study	8,431	50.6	Alcohol	Death	1.9 (1.40–2.70)	5
Lampalo et al. (7)	Croatia	Prospective cohort study	464	50	Alcohol	Relapse	3.46 (1.94–6.11)	8
Peltzer et al. (10)	South Africa	Cross-sectional study	2,670 (men)	39.5	Alcohol	Relapse	1.3 (1.03–1.65)	5
			2,230 (women)	39.5	Alcohol	Relapse	1.44 (1.05–1.97)	
Bartholomay et al. (21)	Brazil	Cohort study	980	37	Alcohol	Death	1.38 (0.88–2.14)	5

OR, odds ratio; CI, confidence interval.

**Figure 2:**
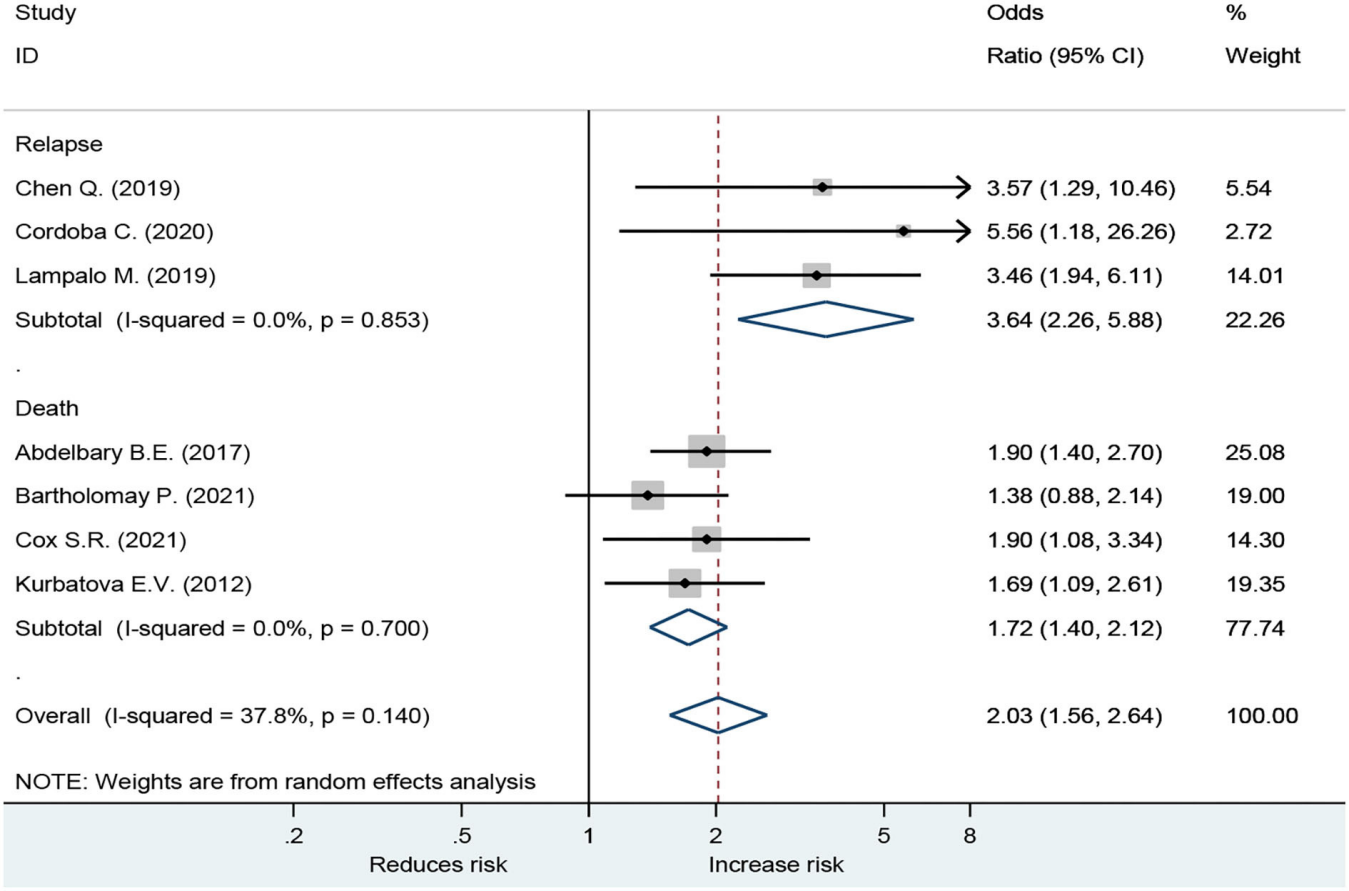
Forest plot showing odds ratio of tuberculosis relapse and death among drinkers.

**Figure 3:**
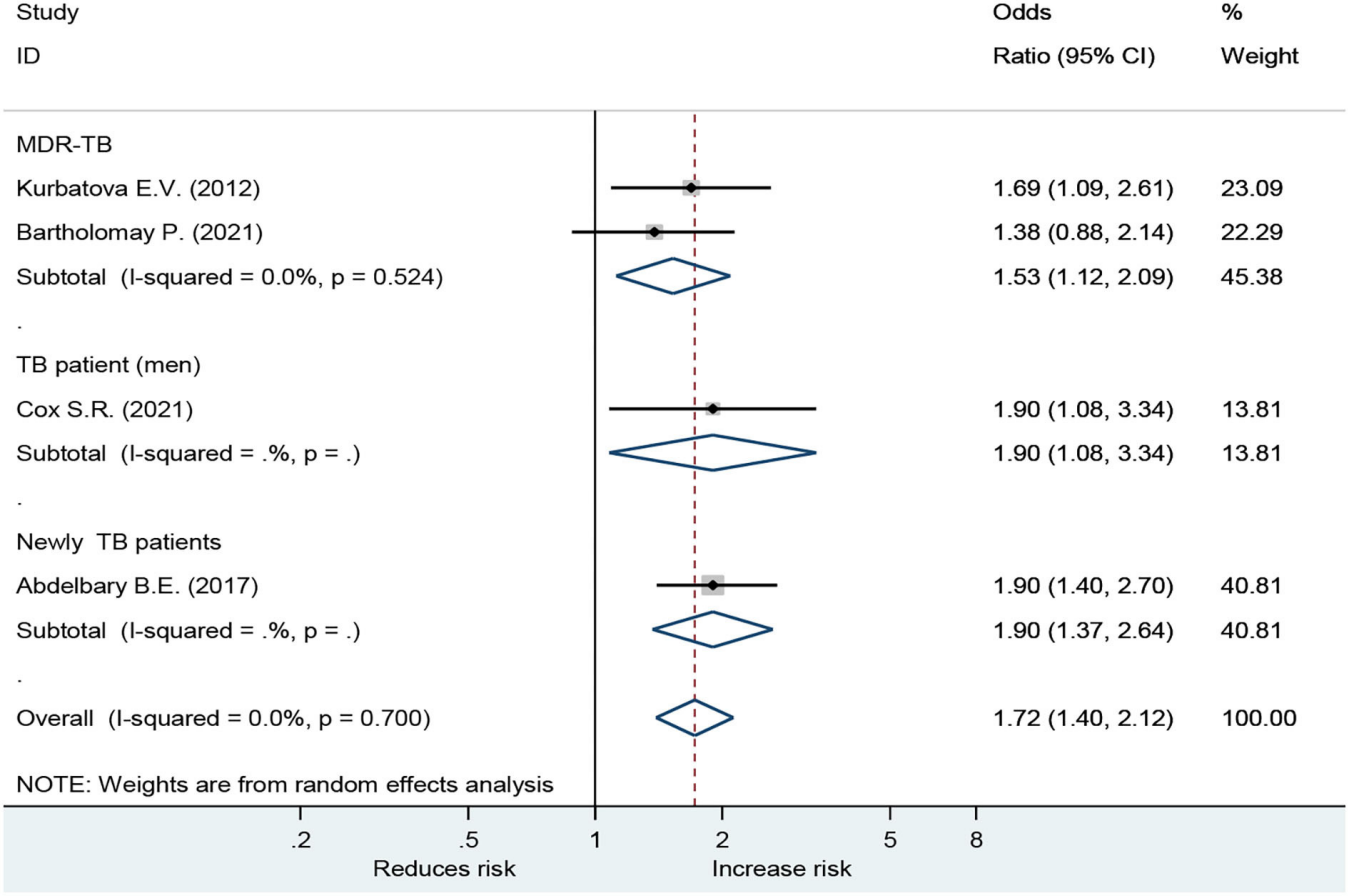
Forest plot showing the effect of alcohol drinkers by tuberculosis type.

Forest plot showed an odds ratio of TB relapse and death among TB patient drinkers. We excluded the study of Peltzer et al. (10) since *I*^2^ was high (73.2%), according to the difference in research method, setting, and age. The results showed that drinking alcohol significantly increased the risk of relapse (OR = 3.64; 95% CI: 2.26–5.88, *p* < 0.001) and also significantly increased the risk of death (OR = 1.72; 95% CI: 1.40–2.12, *p* < 0.001; Figure 2). In the subgroup analysis among drinkers by TB type, the results showed that drinking alcohol in multidrug resistance patients significantly increased the risk of death (OR = 1.53; 95% CI: 1.12–2.09, *p* < 0.001), drinking alcohol in men TB patients significantly increased the risk of death (OR = 1.90; 95% CI: 1.08–3.34, *p* < 0.001), and drinking alcohol in new TB patients significantly increased the risk of death (OR = 1.72; 95% CI: 1.40–2.12, *p* < 0.001) (Figure 3).

The results of the subgroups were similar to those of the overall research (Table 2). Subgroup analyses were conducted using omitted age ≥60 years, omitted MDR TB, and omitted Quality of the study (NOS) Stars <7. For the disease severity among drinkers, for the death among drinkers, and for omitted Age ≥ 60 years, the OR for the random-effects model in the different age groups was 1.72 (95% CI: 1.40–2.12, *p* < 0.001). For relapse among drinkers, the OR for the random-effects model in the different age groups was 3.66 (95% CI: 2.14–6.27, *p* < 0.001). The OR for the random-effects model in the MDR TB for death was 1.53 (95% CI: 1.12–2.09, *p* < 0.001). While the death OR from the random-effects model in the stars ≥5 group (NOS quality of study) was 1.90 (95% CI: 1.08–3.37, *p* < 0.001) (Table 2).

**Table 2:** Sensitivity and subgroup analysis.

**Characteristics**	**Relapse**			**Death**		
	**OR (95% CI)**	**Heterogeneity**		**OR (95% CI)**	**Heterogeneity**	
		***I*^2^ (%)**	***P***		***I*^2^ (%)**	***P***
**Age**						
≥60 years	3.57 (1.29–10.46)	0	<0.001	N/A	N/A	N/A
<60 years	3.66 (2.14–6.27)	0	<0.001	1.72 (1.40–2.12)	0	<0.001
**TB type**						
MDR-TB	N/A	N/A	N/A	1.53 (1.12–2.09)	0	<0.001
TB patient (men)	N/A	N/A	N/A	1.90 (1.08–3.34)	0	<0.001
Newly TB patient	N/A	N/A	N/A	1.90 (1.37–2.64)	0	<0.001
**Quality of the study (NOS) stars**						
≥5	3.64 (2.26–5.88)	0	<0.001	1.90 (1.08–3.37)	0	<0.001
<5	N/A	N/A	N/A	1.70 (1.35–2.12)	0	<0.001

OR, odds ratio; CI, confidence interval; N/A, not available; NOS, Newcastle-Ottawa Scale.

**Figure 4:**
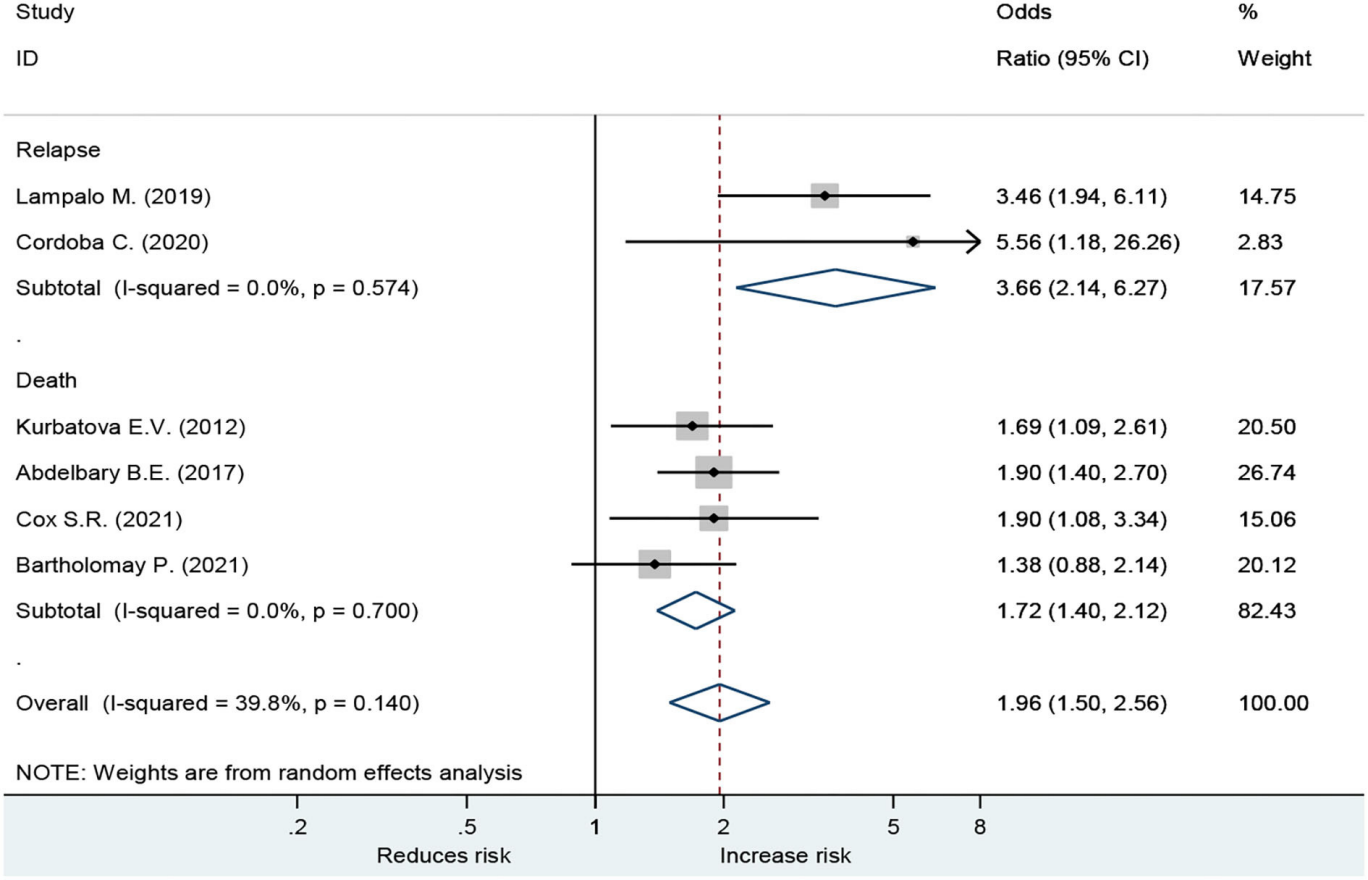
Forest plot showing effect of alcohol on TB relapse in people age < 60 years.

For death outcome, subgroup analysis among drinkers by TB types, the OR for MDR-TB was 1.53 (95% CI: 1.12–2.09, *p* < 0.001). Men had a higher risk with an OR of 1.90 (95% CI: 1.08–3.34, *p* < 0.001) than those in women. In addition, new TB patients were associated with death with OR of 1.90 (95% CI: 1.37–2.64, *p* < 0.001) (Figure 3). A sensitivity analysis found that, when removing the elderly, paper 3, drinking caused relapse by 3.66 (95% CI: 2.14–6.27, *p* < 0.001) (Figure 4).

### Publication bias of included studies

A review of publication bias was carried out. The symmetric funnel plot demonstrated no apparent publication bias; and Begg's and Egger's tests found no significant differences in all age groups and outcomes (Supplementary Figures 1, 2).

## Discussion

To the best of our knowledge, this is the first study to find the relationship between drinking alcohol and relapse in TB patients. We discovered that consuming alcohol substantially increases the risk of relapse in TB patients (OR = 3.64; 95%CI: 2.26–5.88, *p* < 0.001), as well as the risk of mortality (OR = 1.72; 95%CI: 1.40–2.12, *p* < 0.001).

Alcohol is widely consumed by individuals of all ages worldwide. The question of whether drinking is healthy or bad for one's health is always a topic of discussion. Alcohol users may have poorer TB treatment outcomes due to behavioral processes such as poor drug adherence and a higher rate of loss to follow-up (LTFU) (18, 22, 23), or physiological mechanisms, such as how alcohol affects innate and adaptive immune responses (24) lung function and barrier protection (25), hepatotoxicity (26), and TB and human immunodeficiency virus (HIV) drug absorption and metabolism (27). As a result, TB patients experience relapse and death.

Mechanisms of alcohol causing TB relapse can be explained as follows: alcohol reduces the activity of many types of white blood cells in the group of T cells, such as macrophages, CD4+ lymphocytes, and CD8+ lymphocytes. Drinkers have a poorer immune system than the general population. The efficiency of the major medicine used to treat TB, isoniazid, is reduced when alcohol is consumed (9). Relapsed TB has a variety of consequences, including related health diseases, death, loss of income for families, and the state of having to spend a significant amount of money on medical care. Relapse rates are higher, which is associated with heavy drinking or with alcohol use disorder (AUD) behaviors. This appears to support previous findings of a deleterious impact of excessive drinking/AUD on the clinical course of TB, increased relapse rates, and exposure to the most harmful forms of the disease (9, 28), in line with other research (29).

The death of TB patients is a major reason why TB treatment initiatives have failed to reach their objectives. The use of alcohol was linked to a significantly increased risk of death (30). Previous research has looked into the negative consequences of alcohol use and TB, such as death (18–21, 30), treatment failure (10, 22, 30), default (10, 18), and loss of follow-up (30). The answer is in the relapse studies; however, it is still unclear. Relapse has numerous challenges and ramifications for both the patient's family and the healthcare system. The rate of TB relapse has been gradually increasing. As a result, understanding the mechanism, difficulties, and effects of relapse is beneficial to healthcare professionals in treating and caring for patients, as well as approaches for preventing TB relapse.

Selassie et al. (6) found a nearly 4-fold increase in the likelihood of relapse in the group of alcoholics. The Batista study discovered no link between alcohol use and relapse (31) when calculating the amount of alcohol consumed that influences TB. The majority of research did not specify how much drinking affects TB. However, heavy drinkers (20 g or more per day) were shown to be at 1.34 times at risk of TB failure and default in studies (10). The immune system is weakened by alcohol consumption, which increases the risk of active TB. Drinking (~1 to 4 glasses per week) in middle-aged and older adults was connected to a lower risk of dying from various diseases than not drinking (32). However, according to some studies, the amount of drinking formerly suggested for women was one drink per day or fewer, while for males, it was two drinks per day or less. The amount of alcohol consumed is excessive, posing a health risk. Alcohol intake can have a negative impact on one's overall health (33). People who use more than 40 g of alcohol per day and/or have an alcohol use disorder are at a higher risk of developing active TB. This could be due to both an increased risk of infection linked with specific social mixing patterns associated with alcohol use, as well as alcohol's and alcohol-related diseases' influence on the immune system (28). TB relapse patient was associated with alcohol use. Other studies found that TB retreatment patients category (34) and TB medication non-adherence (35, 36) have led to relapses, to be associated with alcohol use. This seems to confirm evidence of AUD on the outcome course of TB, higher relapse rates, and experiencing the most destructive forms of TB (10).

Tuberculosis type and severity of TB patient drinkers are two factors to be considered. The study discovered variables of AUD among patients with MDR-TB compared to their non-TB controls, as well as its connection with disease patterns and associated medical comorbidities. AUD was common in MDR-TB patients and was linked to several medical comorbidities as well as a worsening of the illness's severity (37). Drinking alcohol is a risk factor for relapse and mortality in TB patients, according to our investigation using a large sample size.

## Strengths and limitations

This study offers various advantages. First, we conducted a thorough search of major databases including (CINAHL, PubMed, and Scopus), as is customary when doing a systematic review. Second, we used a broad search approach with no constraints on language or research design. Third, this meta-analysis follows the PRISMA checklist's recommended technique for systematic reviews and meta-analyses. Fourth, our investigation included up-to-date evidence and was carried out utilizing proper statistical procedures for analysis. Finally, the robustness analysis, which included sensitivity analysis and subgroup analysis, demonstrated that the results remained unaltered. The limitations of the study are as follows: First, we searched through four large databases, which may or may not have included all relevant research. Despite this, we discovered no indication of publication bias after using Begg's test, Egger's test, and a funnel plot. Second, this study did not include data on the amount of alcohol consumed, despite the fact that studies have shown that this is an effective method of drinking after being diagnosed.

As a result, the findings should be regarded with care. However, the effects of drinking alcohol in our research were constant across trials, which might imply that the findings are highly generalizable to any situation. Second, the degree of alcohol use was an essential component that may have an impact on our findings. Nonetheless, our sensitivity analysis revealed that drinking alcohol had a detrimental impact on the outcomes.

## Further research directions

To answer issues concerning the likelihood of recurrence and TB patients, well-designed prospective cohort studies are required. There is a need for more data from drinking alcohol that is routinely documented and examined among TB patients. Some characteristics, such as alcohol consumption level, kind and degree of consumption, length of drinking, alcohol addiction, size, amount, and duration of drinking, determine the outcome and determine when to stop drinking. These have an influence on the development of the condition.

## Conclusion

Alcohol use has been identified as a risk factor for TB recurrence and mortality in TB patients. People who consume alcohol have a greater risk of relapse and mortality from TB than those who do not consume alcohol. As a result, initiatives to minimize alcohol use among TB patients should be conducted to reduce the detrimental impacts that may occur to people, families, and communities. The results of this study can be used to inform even clinical practitioners or the policy makers on the control of alcohol consumption in TB patients and the intervention to prevent the recurrence of TB.

## Data availability statement

The original contributions presented in the study are included in the article/Supplementary material, further inquiries can be directed to the corresponding author/s.

## Author contributions

DW and SS: conceptualization. DW, JP, and AU: methodology, formal analysis, and investigation. DW and SP: writing—original draft preparation. JP, SS, and AU: writing—review and editing and supervision. SS: funding acquisition. SP, DW, JP, and AU: resources. All authors contributed to the article and approved the submitted version.

## Funding

For the research, authoring, and/or publishing of this article, the author(s) received a grant from the University of Phayao's School of Pharmaceutical Sciences' Unit of Excellence on Clinical Outcomes Research and Integration (UNICORN).

## Conflict of interest

The authors declare that the research was conducted in the absence of any commercial or financial relationships that could be construed as a potential conflict of interest.

## Publisher's note

All claims expressed in this article are solely those of the authors and do not necessarily represent those of their affiliated organizations, or those of the publisher, the editors and the reviewers. Any product that may be evaluated in this article, or claim that may be made by its manufacturer, is not guaranteed or endorsed by the publisher.

## References

[1] World Health Organization. Global Tuberculosis Report (2021). Available online at: https://www.who.int/publications/digital/global-tuberculosis-report-2021 (accessed March 19, 2022).

[2] World Health Organization. Global Tuberculosis Report 2020 (2020). Available online at: https://www.who.int/publications/i/item/9789240013131 (accessed March 9, 2022).

[3] Chen Q Peng FL Xiong CG Luo D Peng PY Zou JL . Recurrence is a noticeable cause of rifampicin-resistant Mycobacterium tuberculosis in the elderly in Jiangxi, China. Front Public Health. (2019) 7:1–8. 10.3389/fpubh.2019.00182 31380332 PMC6659409

[4] Córdoba C Buriticá PA Pacheco R. Risk factors associated with pulmonary tuberculosis relapses in Cali, Colombia. Biomédica. (2020) 40:102–14. 10.7705/biomedica.5061 32463612 PMC7449113

[5] Riekstinia V Torp L Leimane V. Risk factors for early relapse of tuberculosis in Latvia. Probl Tuberk Bolezn Legk. (2005) 43–7. 15801638

[6] Selassie AW Pozsik C Wilson D Ferguson PL. Why pulmonary tuberculosis recurs: a population-based epidemiological study. Ann Epidemiol. (2005) 15:519–25. 10.1016/j.annepidem.2005.03.002 15921928

[7] Lampalo M Jukić I Bingulac-Popović J Stanić HS Barišić B Popović-Grle S. The role of cigarette smoking and alcohol consumption in pulmonary tuberculosis development and recurrence. Acta Clin Croat. (2019) 58:590–4. 10.20471/acc.2019.58.04.04 32595242 PMC7314290

[8] Patel KR Patel A Gadhiya NB. Risk factors for sputum positive pulmonary tuberculosis retreatment cases and factors responsible for treatment outcome. J Assoc Physicians India. (2019) 67:56–8. 31562718

[9] Rehm J Samokhvalov AV Neuman MG Room R Parry C Lönnroth K . The association between alcohol use, alcohol use disorders and tuberculosis (TB). A systematic review. BMC Public Health. (2009) 9:1–12. 10.1186/1471-2458-9-450 19961618 PMC2796667

[10] Peltzer K Louw J Mchunu G Naidoo P Matseke G Tutshana B. Hazardous and harmful alcohol use and associated factors in tuberculosis public primary care patients in South Africa. Int J Environ Res Public Health. (2012) 9:3245–57. 10.3390/ijerph9093245 23202681 PMC3499864

[11] Liberati A Altman DG Tetzlaff J Mulrow C Gøtzsche PC Ioannidis JPA . The PRISMA statement for reporting systematic reviews and meta-analyses of studies that evaluate healthcare interventions: explanation and elaboration. BMJ. (2009) 339:b2700. 10.1136/bmj.b2700 19622552 PMC2714672

[12] Afshar B Carless J Roche A Balasegaram S Anderson C. Surveillance of tuberculosis (TB) cases attributable to relapse or reinfection in London, 2002-2015. PLoS ONE. (2019) 14:1–12. 10.1371/journal.pone.0211972 30768624 PMC6377187

[13] Higgins JPT Thompson SG Deeks JJ Altman DG. Measuring inconsistency in meta-analyses. BMJ. (2003) 327:557–60. 10.1136/bmj.327.7414.557 12958120 PMC192859

[14] Begg CB Berlin JA. Publication bias and dissemination of clinical research. J Natl Cancer Inst. (1989) 81:107–15. 10.1093/jnci/81.2.107 2642556

[15] Sterne JAC Egger M. Funnel plots for detecting bias in meta-analysis: guidelines on choice of axis. J Clin Epidemiol. (2001) 54:1046–55. 10.1016/S0895-4356(01)00377-8 11576817

[16] Bahl A Van Baalen MN Ortiz L Chen NW Todd C Milad M . Early predictors of in-hospital mortality in patients with COVID-19 in a large American cohort. Intern Emerg Med. (2020) 15:1485–99. 10.1007/s11739-020-02509-7 32970246 PMC7512216

[17] Duval S Tweedie R. Trim and fill: A simple funnel-plot-based method of testing and adjusting for publication bias in meta-analysis. Biometrics. (2000) 56:455–63. 10.1111/j.0006-341X.2000.00455.x 10877304

[18] Kurbatova EV Taylor A Gammino VM Bayona J Becerra M Danilovitz M . Predictors of poor outcomes among patients treated for multidrug-resistant tuberculosis at DOTS-plus projects. Tuberculosis. (2012) 92:397–403. 10.1016/j.tube.2012.06.003 22789497 PMC4749016

[19] Cox SR Gupte AN Thomas B Gaikwad S Mave V Padmapriyadarsini C . Unhealthy alcohol use independently associated with unfavorable TB treatment outcomes among Indian men. Int J Tubercul Lung Dis. (2021) 25:182–90. 10.5588/ijtld.20.0778 33688806 PMC13404431

[20] Abdelbary BE Garcia-Viveros M Ramirez-Oropesa H Rahbar MH Restrepo BI. Predicting treatment failure, death and drug resistance using a computed risk score among newly diagnosed TB patients in Tamaulipas, Mexico. Epidemiol Infect. (2017) 145:3020–34. 10.1017/S0950268817001911 28903800 PMC9152743

[21] Bartholomay P Pinheiro RS Dockhorn F Pelissari DM de Araújo WN. Brazilian cohort study of risk factors associated with unsuccessful outcomes of drug resistant tuberculosis. BMC Infect Dis. (2021) 21:1–13. 10.1186/s12879-021-06756-7 34627179 PMC8502313

[22] De Albuquerque MDFPM Ximenes RADA Lucena-Silva N De Souza WV Dantas AT Dantas OMS . Factors associated with treatment failure, dropout, and death in a cohort of tuberculosis patients in Recife, Pernambuco State, Brazil. Cad Saude Publica. (2007) 23:1573–82. 10.1590/S0102-311X2007000700008 17572806

[23] Miller AC Gelmanova IY Keshavjee S Atwood S Yanova G Mishustin S . Alcohol use and the management of multidrug-resistant tuberculosis in Tomsk, Russian Federation. Int J Tubercul Lung Dis. (2012) 16:891–6. 10.5588/ijtld.11.0795 22507895 PMC8324013

[24] Molina PE Happel KI Zhang P Kolls JK Nelson S. Focus on: Alcohol and the immune system. Alcohol Res Health. (2010) 33:97–108. 23579940 PMC3887500

[25] Quintero D Guidot DM. Focus on the lung. Alcohol Res Health. (2010) 33:219–28. 23584063 PMC3860507

[26] Saukkonen JJ Cohn DL Jasmer RM Schenker S Jereb JA Nolan C . An official ATS statement: hepatotoxicity of antituberculosis therapy. Am J Respir Crit Care Med. (2006) 174:935–52. 10.1164/rccm.200510-1666ST 17021358

[27] Williams EC Hahn JA Saitz R Bryant K Lira MC Samet JH. Alcohol use and human immunodeficiency virus (HIV) Infection: current knowledge, implications, and future directions. Alcohol Clin Exp Res. (2016) 40:2056–72. 10.1111/acer.13204 27696523 PMC5119641

[28] Lönnroth K Williams BG Stadlin S Jaramillo E Dye C. Alcohol use as a risk factor for tuberculosis - A systematic review. BMC Public Health. (2008) 8:1–12. 10.1186/1471-2458-8-289 18702821 PMC2533327

[29] Paixão LMM Gontijo ED. Profile of notified tuberculosis cases and factors associated with treatment dropout. Rev Saude Publica. (2007) 41:205–13. 10.1590/S0034-89102007000200006 17384794

[30] Ragan EJ Kleinman MB Sweigart B Gnatienko N Parry CD Horsburgh CR . The impact of alcohol use on tuberculosis treatment outcomes: asystematic review and meta-analysis. Int J Tubercul Lung Dis. (2020) 24:73–82. 10.5588/ijtld.19.0080 32005309 PMC7491444

[31] Batista JDAL Militão De Albuquerque MFP De Alencar Ximenes RA Rodrigues LC. Smoking increases the risk of relapse after successful tuberculosis treatment. Int J Epidemiol. (2008) 37:841–51. 10.1093/ije/dyn113 18556729 PMC2483312

[32] Kunzmann AT Coleman HG Huang WY Berndt SI. The association of lifetime alcohol use with mortality and cancer risk in older adults: a cohort study. PLoS Med. (2018) 15:1–18. 10.1371/journal.pmed.1002585 29920516 PMC6007830

[33] Wood AM Kaptoge S Butterworth A Nietert PJ Warnakula S Bolton T . Risk thresholds for alcohol consumption: combined analysis of individual-participant data for 599 912 current drinkers in 83 prospective studies. Lancet. (2018) 391:1513–23. 10.1016/S0140-6736(18)30134-X 29676281 PMC5899998

[34] Suhadev M Thomas BE Raja Sakthivel M Murugesan P Chandrasekaran V Charles N . Alcohol use disorders (AUD) among tuberculosis patients: a study fromChennai, South India. PLoS ONE. (2011) 6:1–6. 10.1371/journal.pone.0019485 21611189 PMC3096635

[35] Shin SS Mathew TA Yanova GV Fitzmaurice GM Livchits V Yanov SA . Alcohol consumption among men and women with tuberculosis in Tomsk, Russia. Cent Eur J Public Health. (2010) 18:132–8. 10.21101/cejph.a3590 21033607 PMC3062936

[36] Muture BN Keraka MN Kimuu PK Kabiru EW Ombeka VO Oguya F. Factors associated with default from treatment among tuberculosis patients in nairobi province, Kenya: a case control study. BMC Public Health. (2011) 11:1–10. 10.1186/1471-2458-11-696 21906291 PMC3224095

[37] Lasebikan VO Ige OM. Alcohol use disorders in multidrug resistant tuberculosis (MDR-TB) patients and their non-tuberculosis family contacts in Nigeria. Pan Afr Med J. (2020) 36:1–14. 10.11604/pamj.2020.36.321.17118 33193975 PMC7603812

