# Supplementary material for: Effect of alcohol consumption on relapse outcomes among tuberculosis patients: A systematic review and meta-analysis
Source: Front Public Health. 2022 Nov 3;10:962809. doi: 10.3389/fpubh.2022.962809 (PMC9669980; doi:10.3389/fpubh.2022.962809)
Supplement: Supplementary file 1 [file Data_Sheet_1.pdf]

# *Supplementary Material*

**Effect of alcohol consumption on relapse outcomes among tuberculosis patients: A systematic review and meta-analysis**

**Running title**: Alcohol consumption and tuberculosis relapse

**Dao Weiangkham[1], Adinat Umnuaypornlert[2], Surasak Saokaew[3], Samrerng Prommongkol[4], Jutamas Ponmark[5]***

[1] Department of Nursing, School of Nursing, University of Phayao, Phayao, Thailand.

[2] Department of Pharmaceutical Care, School of Pharmaceutical Sciences, University of Phayao, Phayao, Thailand.

[3] Department of Pharmaceutical Care, School of Pharmaceutical Sciences, University of Phayao, Phayao, Thailand.

[4] Mahidol Bangkok School of Tropical Medicine (Mahidol-BSTM), Faculty of Tropical medicine, Mahidol University, Thailand.

[5] Department of Nursing, School of Nursing, University of Phayao, Phayao, Thailand.

***Correspondence:**

Dr. Jutamas Ponmark[5], Ph.D.

Department of Nursing, School of Nursing, University of Phayao, Phayao, Thailand.

Tel: +66882604056 ; Email:Jutamasponmark@gmail.com

**Supplementary data**

**Table**
Table S1. Search algorithms.
Table S2. Risk of bias assessment of cohort studies included in the meta-analysis by the Newcastle-Ottawa Scale.
Table S3. Description of outcomes in studies included in A meta-analysis.
Table S4. Full-text articles excluded.

**Figure**
Figure S1. Funnel plot of relapse
Figure S2. Funnel plot of death

**Table S1.** Search algorithms

| Database | No | Step search algorithm | Items found |
|---|---|---|---|
| Pubmed | #1 | Search tuberculosis | 274,279 |
| | #2 | Search "alcohol consumption" | 46,884 |
| | #3 | Search "alcohol consumption" OR "alcohol drinking" | 99,357 |
| | #4 | Search "relapse" | 669,560 |
| | #5 | Search "relapse" OR "reinfection" OR "recurrence" OR "retreatment" OR "failure" | 1,556,658 |
| | #6 | Search (((tuberculosis) AND (("alcohol consumption)" OR "alcohol drinking") AND ("relapse" OR "reinfection" OR "recurrence" OR "retreatment" OR "failure") | 49 |
| Scopus | #1 | Search tuberculosis | 639,055 |
| | #2 | Search "alcohol consumption" | 249,986 |
| | #3 | Search "alcohol consumption" OR "alcohol drinking" | 283,503 |
| | #4 | Search "relapse" | 508,949 |
| | #5 | Search "relapse" OR "reinfection" OR "recurrence" OR "retreatment" OR "failure" | 6,120,287 |
| | #6 | Search (((tuberculosis) AND (("alcohol consumption)" OR "alcohol drinking") AND ("relapse" OR "reinfection" OR "recurrence" OR "retreatment" OR "failure") | 1,454 |
| Cinahl | #1 | Search tuberculosis | 54,441 |
| | #2 | Search "alcohol consumption" | 41,249 |
| | #3 | Search "alcohol consumption" OR "alcohol drinking" | 63,967 |
| | #4 | Search "relapse" | 62,406 |
| | #5 | Search "relapse" OR "reinfection" OR "recurrence" OR "retreatment" OR "failure" | 611,944 |
| | #6 | Search (((tuberculosis) AND (("alcohol consumption)" OR "alcohol drinking") AND ("relapse" OR "reinfection" OR "recurrence" OR "retreatment" OR "failure") | 610 |

**Table S2.** Risk of bias assessment of 8 studies included in the meta-analysis by the Newcastle-Ottawa Scale

| **No.** | **Author (Year)** | **Adequacy selection of case-control** | | | | **Comparability of studies** | | **Outcome assessment** | | | **Total NOS score** |
|---|---|---|---|---|---|---|---|---|---|---|---|
| | | Is the case definition adequate? | Representativeness of the cases | Selection of Controls | Definition of Controls | Study control for Age, tb type of tuberculosis | Additional factors; controlled for ≥ 2 variables including Characteristic factors | Ascertainment of exposure | Same method of ascertainment for cases and controls | Non-Response rate | |
| 1 | Chen et al. (2019) | ֍ | ֍ | ֍ | ֍ | ֍ | ֍ | ֍ | ֍ | - | 8/9 |
| 2 | Cordoba et al. (2020) | ֍ | ֍ | ֍ | ֍ | ֍ | ֍ | ֍ | ֍ | - | 8/9 |
| 3 | Kurbatova et al. (2012) | ֍ | ֍ | - | - | ֍ | ֍ | ֍ | - | - | 5/9 |
| 4 | Cox et al. (2021) | ֍ | ֍ | ֍ | ֍ | ֍ | - | ֍ | ֍ | - | 8/9 |
| 5 | Abdelbary et al. (2017) | ֍ | ֍ | - | - | ֍ | ֍ | ֍ | - | - | 5/9 |
| 6 | Lampalo et al. (2019) | ֍ | ֍ | ֍ | ֍ | ֍ | ֍ | ֍ | ֍ | - | 8/9 |

| **No.** | **Author (Year)** | **Adequacy selection of case-control** | | | | **Comparability of studies** | | **Outcome assessment** | | | **Total NOS score** |
|---|---|---|---|---|---|---|---|---|---|---|---|
| | | Is the case definition adequate ? | Representativeness of the cases | Selection of Controls | Definition of Controls | Study control for Age, tb type of tuberculosis | Additional factors; controlled for ≥ 2 variables including Characteristic factors | Ascertainment of exposure | Same method of ascertainment for cases and controls | Non-Response rate | |
| 7 | Peltzer et al. (2012) | ֍ | ֍ | - | - | ֍ | ֍ | ֍ | - | - | 5/9 |
| 8 | Bartholomay et al. (2021) | ֍ | ֍ | - | - | ֍ | ֍ | ֍ | - | - | 5/9 |

**Table S3.** Description of outcomes in studies included in meta-analysis

| Author (Year) | Outcomes | Definitions |
|---|---|---|
| Chen et al., 2019 | Failure/ dropout/ death | Recurrent patients in this study met the criteria previously reported in the literature. Essentially, there are two aspects to the criteria for recurrence:(1) completion of the standard anti-TB treatment process and (2) more than two episodes of diagnosed TB (within a minimum time interval of 12 months based on the date of the end of treatment for the first episode). Non-recurrent TB cases are defined as TB patients who test positive for the first time. Relapse, known as endogenous reactivation, is caused by the same strain that caused the first episode of TB. |
| Cordoba et al., 2020 | relapse | The relapse cases were defined as those patients readmitted to the control program due to relapse as defined by the World Health Organization (WHO). A patient previously treated for tuberculosis was declared as cured based on three successive negative smears or negative cultures in the four months after treatment. Controls were defined as those with a first episode of tuberculosis diagnosed in the same year as the case and with no relapse after the same follow-up period. |
| Kurbatova et al., 2012 | Death/ failure/ default | Baseline drug resistance was determined by drug susceptibility tests (DST) in the local laboratory from sputum collected between 60 days prior to 30 days after MDR-TB treatment initiation. Extensively drug-resistant (XDR) TB and SLD groups were defined according WHO guidelines. |
| Cox et al., 2021 | failure / recurrence/ Death | TB treatment outcomes included failure, recurrence or death.<br><br>Treatment failure was defined as testing positive for TB on sputum microscopy or culture during the last 2 months of treatment.<br><br>TB recurrence was defined as testing positive for TB on sputum microscopy or culture following successful treatment completion.<br><br>Death was defined as all-cause mortality within 24 months of treatment initiation. |

| Author (Year) | Outcomes | Definitions |
|---|---|---|
| Abdelbary et al., 2017 | treatment failure, death, and drug resistance (all as binary outcomes). | Treatment failure indicated smear-positive persistence at or after 5 months of treatment with first-line anti-TB medications. Death included all causes of death (TB or non-TB related) during the course of TB treatment.<br><br>Drug resistance was evaluated for five antibiotics (isoniazid, rifampin, pyrazinamide, streptomycin, and ethambutol) using culture methods and coded as present if resistance to any of the five drugs was detected. Multi-drug resistance (MDR) included resistance to isoniazid and rifampicin**.** |
| Lampalo et al., 2019 | Tb relapse | - |
| Peltzer et al., 2012 | Tb relapse | A health care provider who identified a new TB treatment or retreatment patient (within one month on<br><br>treatment) |
| Bartholomay et al., 2021 | Failure | Failure was defined in the following situations: persistence of smear positive sputum at the end of treatment; cases who had strongly positive smear (++or+ ++) at the beginning of treatment and maintained this situation until the fourth month; initial positive smear followed by negative; and new positive results for two consecutive months, from the fourth month of treatment |
| | Death | Cases that died from tuberculosis or from other causes during the treatment were considered in the outcome death. |

**Table S4.** Full-text articles excluded

| | Author, Year | Reason for exclusion | | Author, Year | Reason for exclusion |
|---|---|---|---|---|---|
| 1 | Yen et al., 2017 | No outcome of interest | 12 | Duraisamy et al., 2014 | No outcome of interest |
| 2 | Shin et al., 2010 | No outcome of interest | 13 | De Albuquerque et al., 2007 | No outcome of interest |
| 3 | Karl Peltzer et al., 2013 | No outcome of interest | 14 | Ragan et al., 2020 | No outcome of interest |
| 4 | Peltzer., 2014 | Other study design | 15 | Rehm et al., 2009 | Other study design |
| 5 | Myers et al., 2018 | No outcome of interest | 16 | Peltzer et al., 2012 | Other study design |
| 6 | Mulu et al., 2015 | No outcome of interest | 17 | Peltzer and Louw, 2014 | Inappropriate population |
| 7 | Montiel et al., 2020 | Insufficient information | 18 | Salami and Oluboyo, 2003 | No outcome of interest |
| 8 | Shin et al., 2013 | Other study design | 19 | De Albuquerque et al., 2001 | Other study design |
| 9 | Ma, Du, et al., 2019 | Other study design | 20 | dos Santos et al., 2021 | No outcome of interest |
| 10 | Ma, Che, et al., 2019 | No outcome of interest | 21 | Necho et al., 2021 | No outcome of interest |
| 11 | Louwagie et al., 2020 | Other study design | 22 | Chenciner et al., 2021 | Other study design |

# Figures

**Figure S1.** Funnel plot of relapse

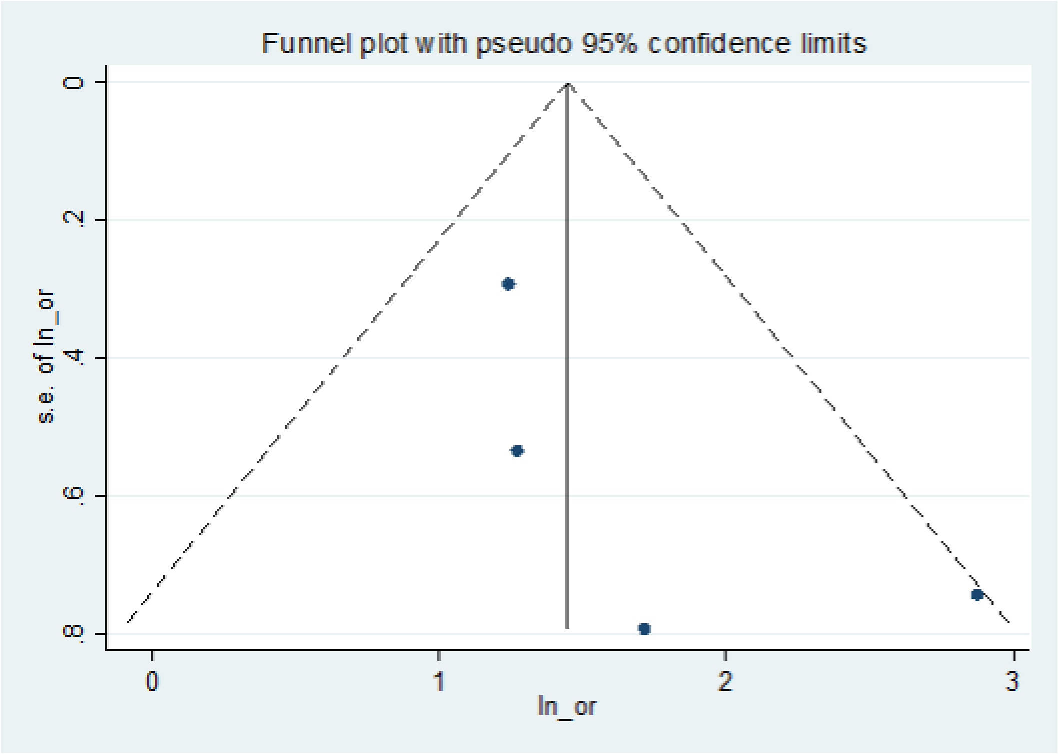


**Figure S2.** Funnel plot of death

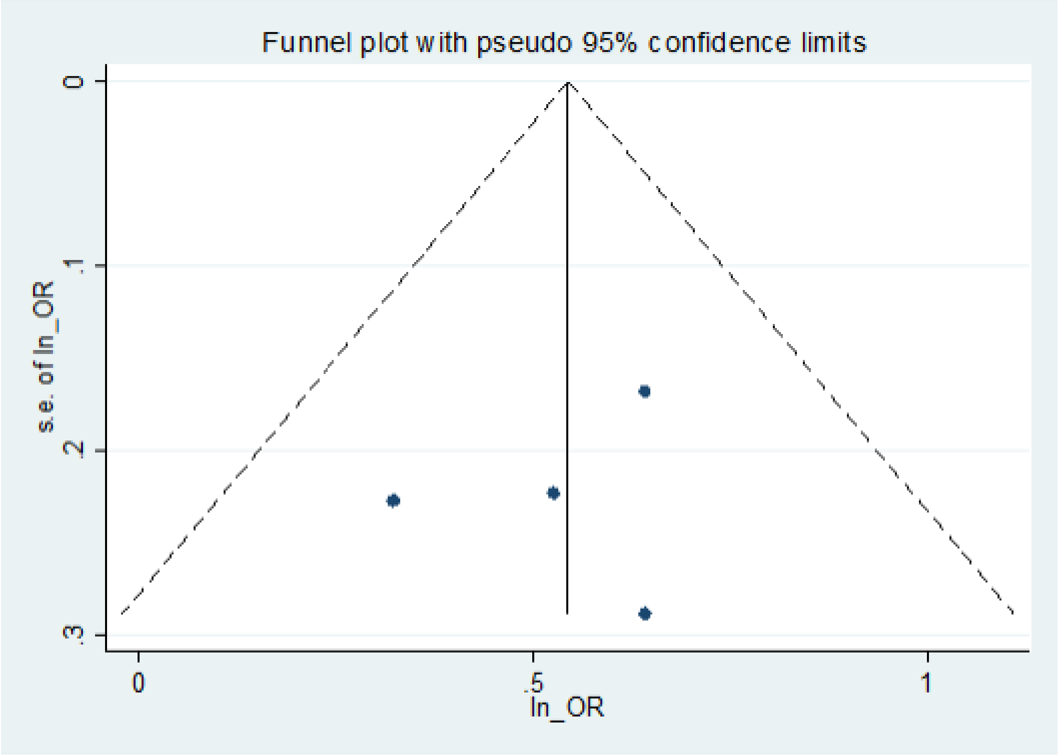

**Reference**

De Albuquerque, M. D. F. M. *et al.* (2001) '[Prognostic factors for pulmonary tuberculosis outcome in Recife, Pernambuco, Brazil]', *Revista panamericana de salud publica = Pan American journal of public health*, 9(6), pp. 368–374. doi: 10.1590/S1020-49892001000600003.

De Albuquerque, M. D. F. P. M. *et al.* (2007) 'Factors associated with treatment failure, dropout, and death in a cohort of tuberculosis patients in Recife, Pernambuco State, Brazil', *Cadernos de Saúde Pública*, 23(7), pp. 1573–1582. doi: 10.1590/S0102-311X2007000700008.

Chenciner, L. *et al.* (2021) 'Social and health factors associated with unfavourable treatment outcome in adolescents and young adults with tuberculosis in Brazil: a national retrospective cohort study', *The Lancet. Global health*, 9(10), pp. e1380–e1390. doi: 10.1016/S2214-109X(21)00300-4.

*Conjoint alcohol and tobacco use among tuberculosis patients in public primary healthcare in South Africa* (no date). Available at: http://www.scielo.org.za/scielo.php?script=sci_abstract&pid=S2078-67862014000100005&lng=en&nrm=iso (Accessed: 3 May 2022).

Duraisamy, K. *et al.* (2014) 'Does Alcohol consumption during multidrug-resistant tuberculosis treatment affect outcome?. A population-based study in Kerala, India', *Annals of the American Thoracic Society*, 11(5), pp. 712–715. doi: 10.1513/ANNALSATS.201312-447OC.

Louwagie, G. M. *et al.* (2020) 'Addressing tobacco smoking and drinking to improve TB treatment outcomes, in South Africa: a feasibility study of the ProLife program', *Translational behavioral medicine*, 10(6), pp. 1491–1503. doi: 10.1093/TBM/IBZ100.

Ma, Y., Du, J., *et al.* (2019) 'Effect of alcohol drinking on sputum conversion at the end of second month and outcome of smear-positive pulmonary tuberculosis patients', *Zhonghua yi xue za zhi*, 99(14), pp. 1090–1094. doi: 10.3760/CMA.J.ISSN.0376-2491.2019.14.011.

Ma, Y., Che, N. Y., *et al.* (2019) 'The joint impact of smoking plus alcohol drinking on treatment of pulmonary tuberculosis', *European journal of clinical microbiology & infectious diseases : official publication of the European Society of Clinical Microbiology*, 38(4), pp. 651–657. doi: 10.1007/S10096-019-03489-Z.

Montiel, I. *et al.* (2020) 'Factors associated with unsuccessful treatment of patients with drug-sensitive tuberculosis in Paraguay', *Revista Panamericana de Salud Publica/Pan American Journal of Public Health*, 44. doi: 10.26633/RPSP.2020.89.

Mulu, W. *et al.* (2015) 'Risk factors for multidrug resistant tuberculosis patients in Amhara National Regional State', *African health sciences*, 15(2), pp. 368–377. doi: 10.4314/AHS.V15I2.9.

Myers, B. *et al.* (2018) 'Impact of alcohol consumption on tuberculosis treatment outcomes: a prospective longitudinal cohort study protocol', *BMC infectious diseases*, 18(1). doi:

10.1186/S12879-018-3396-Y.

Necho, M. *et al.* (2021) 'Prevalence and associated factors for alcohol use disorder among tuberculosis patients: a systematic review and meta-analysis study', *Substance Abuse: Treatment, Prevention, and Policy*, 16(1), pp. 1–15. doi: 10.1186/S13011-020-00335-W/TABLES/4.

Peltzer, K. *et al.* (2012) 'Hazardous and Harmful Alcohol Use and Associated Factors in Tuberculosis Public Primary Care Patients in South Africa', *International Journal of Environmental Research and Public Health*, 9(9), p. 3245. doi: 10.3390/IJERPH9093245.

Peltzer, K. *et al.* (2013) 'Screening and brief interventions for hazardous and harmful alcohol use among patients with active tuberculosis attending primary public care clinics in South Africa: results from a cluster randomized controlled trial', *BMC Public Health*, 13(1), p. 699. doi: 10.1186/1471-2458-13-699.

Peltzer, K. and Louw, J. S. (2014) 'Prevalence and factors associated with tuberculosis treatment outcome among hazardous or harmful alcohol users in public primary health care in South Africa', *African health sciences*, 14(1). doi: 10.4314/AHS.V14I1.24.

Ragan, E. J. *et al.* (2020) 'The impact of alcohol use on tuberculosis treatment outcomes: a systematic review and meta-analysis', *The international journal of tuberculosis and lung disease : the official journal of the International Union against Tuberculosis and Lung Disease*, 24(1), pp. 73–82. doi: 10.5588/IJTLD.19.0080.

Rehm, J. *et al.* (2009) 'The association between alcohol use, alcohol use disorders and tuberculosis (TB). A systematic review', *BMC Public Health*, 9(1), pp. 1–12. doi: 10.1186/1471-2458-9-450/TABLES/2.

Salami, A. K. and Oluboyo, P. O. (2003) 'Management outcome of pulmonary tuberculosis: a nine year review in Ilorin', *West African journal of medicine*, 22(2), pp. 114–119. doi: 10.4314/WAJM.V22I2.27928.

dos Santos, D. T. *et al.* (2021) 'Survival time among patients who were diagnosed with tuberculosis, the precocious deaths and associated factors in southern Brazil', *Tropical Medicine and Health*, 49(1), pp. 1–14. doi: 10.1186/S41182-021-00320-4/TABLES/5.

Shin, S. *et al.* (2013) 'Effectiveness of Alcohol Treatment Interventions Integrated into Routine Tuberculosis Care in Tomsk, Russia', *Addiction (Abingdon, England)*, 108(8), p. 1387. doi: 10.1111/ADD.12148.

Shin, S. S. *et al.* (2010) 'Alcohol consumption among men and women with tuberculosis in Tomsk, Russia', *Central European journal of public health*, 18(3), p. 132. doi: 10.21101/CEJPH.A3590.

Yen, Y. F. *et al.* (2017) 'Heavy alcohol consumption increases the risk of active tuberculosis in Taiwanese adults: a nation-wide population-based cohort study.', *Addiction (Abingdon, England)*, 112(12), pp. 2124–2131. doi: 10.1111/ADD.13926.
